# Association of *PGC-1alpha *polymorphisms with age of onset and risk of Parkinson's disease

**DOI:** 10.1186/1471-2350-12-69

**Published:** 2011-05-19

**Authors:** Joanne Clark, Sonika Reddy, Kangni Zheng, Rebecca A Betensky, David K Simon

**Affiliations:** 1 Beth Israel Deaconess Medical Center and Harvard Medical School, 330 Brookline Avenue, Room CLS-638, Boston, MA, 02215, USA; 2 Harvard School of Public Health, Bldg 2, Rm 421, 655 Huntington Ave, Boston, MA 02115, USA

## Abstract

**Background:**

Peroxisome proliferator-activated receptor-γ co-activator (PGC)-1α is a transcriptional co-activator of antioxidant genes and a master regulator of mitochondrial biogenesis. Parkinson's disease (PD) is associated with oxidative stress and mitochondrial dysfunction and recent work suggests a role for PGC-1α. We hypothesized that the rs8192678 *PGC-1α *single nucleotide polymorphism (SNP) may influence risk or age of onset of PD. The A10398G mitochondrial SNP has been inversely associated with risk of PD in some studies. In the current study we analyzed whether rs8192678 or other *PGC-1α *SNPs affect PD risk or age of onset, singularly or in association with the A10398G SNP.

**Methods:**

Genomic DNA samples from 378 PD patients and 173 age-matched controls were analyzed by multiplexed probe sequencing, followed by statistical analyses of the association of each SNP, alone or in combination, with risk or age of onset of PD. Adjustments were made for age of onset being less than the age of sampling, and for the observed dependence between these two ages. The PD samples were obtained as two separate cohorts, therefore statistical methods accounted for different sampling methods between the two cohorts, and data were analyzed using Cox regression adjusted for sampling in the risk set definition and in the model.

**Results:**

The rs8192678 PGC-1α SNP was not associated with the risk of PD. However, an association of the *PGC-1α *rs8192678 GG variant with longevity was seen in control subjects (p = 0.019). Exploratory studies indicated that the CC variant of rs6821591 was associated with risk of early onset PD (p = 0.029), with PD age of onset (p = 0.047), and with longevity (p = 0.022). The rs2970848 GG allele was associated with risk of late onset PD (p = 0.027).

**Conclusions:**

These data reveal possible associations of the *PGC-1α *SNPs rs6821591 and rs2970848 with risk or age of onset of PD, and of the *PGC-1α *rs8192678 GG and the rs6821591 CC variants with longevity. If replicated in other datasets, these findings may have important implications regarding the role of *PGC-1α *in PD and longevity.

## Background

Parkinson's disease is the second most common age-related neurodegenerative disease [1]. PD is characterized in part by a loss of dopaminergic neurons in the substantia nigra, which leads to less dopamine release in the striatum and ultimately to impaired physical movement. PD is a complex disorder involving multiple genetic and environmental factors [2]. Mutations in a number of genes including α-synuclein (SNCA), Parkin, Leucine-Rich-Repeat-Kinase 2 (LRRK2), Phosphatase and Tensin (PTEN) Homolog-Induced Putative Kinase 1 (PINK1) and DJ-1 among others can cause PD or increase the risk of PD [3-6]. A mitochondrial complex I NADH dehydrogenase subunit 3 (ND3) allelic variant, 10398G, has been associated with a decreased risk of PD in some studies [7,8], but not in others [9-11].

These studies reveal that several genes that regulate mitochondrial function or oxidative stress also influence the risk of PD [12-14]. This work, along with that outlining the neurological phenotype of the *Pgc-1α *knockout mouse [15], led us to consider *PGC-1α*, which codes for a critical regulator of mitochondrial metabolism, as a candidate susceptibility gene for PD. PGC-1α is a multifunctional protein that activates most nuclear receptors and also functions as a co-activator to many transcription factors with the net result of co-ordinately regulating mitochondrial biogenesis [16]. PGC-1α links metabolic activity to relevant environmental stimuli in multiple pathways including those responsible for adipogenesis, gluconeogenesis, myogenesis and mitogenesis [17]. In addition, PGC-1α can co-ordinate the expression of many antioxidant programs in response to oxidative stress [15,18,19]. PGC-1α is important for protection against MPTP toxicity [15] and levels of PGC-1α are reduced in substantia nigra neurons in PD [20], suggesting a role for PGC-1α in the pathogenesis of PD.

Because PGC-1α is involved with gluconeogenesis, many association studies have focused on the link between polymorphisms in the *PGC-1α *gene and diabetes. A number of studies have implicated the rs8192678 *PGC-1α *G1444A polymorphism, which converts a glycine to a serine (Gly482Ser) and has been linked to impaired PGC-1α activity [21] as well as to glucose intolerance and diabetes [22-29]. A recent study established an additive interaction for the association between increased type 2 diabetes mellitus (T2DM) risk and the *PGC-1α *1444A, *UCP2 *866G and *ND3 *10398A genotypes [30]. Further work has implicated the rs7665116 and rs6821591 non-coding *PGC-1α *polymorphisms with age of onset in Huntington's disease [31,32].

The A10398G transversion in the mitochondrial complex I NADH dehydrogenase subunit 3 (*ND3*) gene changes the penultimate amino acid from alanine to threonine (Thr47Ala) [33]. In some studies the G allele has been associated with a decreased risk of PD [7,8], whereas in other studies no association has been found between the 10398G allelic variant and PD [9,10]. Also, as a mitochondrial DNA (mtDNA) SNP, the ND3 genotype may interact with other genes that influence mitochondrial activity, such as any functional PGC-1α SNPs [30].

Interestingly, patients with PD are more likely to have glucose intolerance [34], and type 2 diabetes is associated with an increased risk of Parkinson's disease [35]. Furthermore, the symptoms of PD are more severe in diabetic individuals [36]. We now report results of our investigation into the association of the rs8192678 *PGC-1α *polymorphism with risk for PD and age of onset of PD, either alone or in combination with the A10398G mtDNA polymorphism and exploratory analyses of the association of 14 other *PGC-1α *SNPs and the A10398G mtDNA polymorphism with PD.

## Methods

### Samples

Genomic DNA samples from human blood samples were obtained from the National Institute of Neurological Disorders and Stroke (NINDS) Human Genetics DNA and Cell Line Repository at the Coriell Institute for Medical Research (Camden, New Jersey). The first, detection cohort of samples (PD Cohort 1) consisted of DNA from 179 Caucasian PD patients. The second, validation cohort of samples (PD Cohort 2) consisted of DNA from 199 Caucasian PD patients with either earlier onset PD (age 35-55 years) or later onset PD (age 63-87 years) and from 173 age-matched Caucasian controls.

The two cohorts of samples were further divided into subgroups for exploratory statistical analyses as follows: PD-ALL: all PD samples from both cohorts; PD-Cohort 1: the original cohort of PD samples, which were not selected for age of onset; PD-Cohort 2: all of the PD samples comprising the second group of samples that were selected for age of onset; PD-EARLY: samples from PD-Cohort 2 with ages of onset between 35 to 55 years; PD-LATE: samples from PD-Cohort 2 with ages of onset between 63 to 87 years; PD-ALL-EARLY: the PD-EARLY samples plus those from PD-Cohort 1 within the 35 to 55 year range; PD-ALL-LATE: the PD-LATE samples plus those from PD-Cohort 1 within the 63 to 87 year range.

### Screening of Samples by sequencing and Polymerase Chain Reaction-Restriction Fragment Length Polymorphism (PCR-RFLP)

The samples were genotyped using Sequenom^®^iPLEX genotyping at the Harvard-Partners Center for Genetics and Genomics Genotyping Facility (Cambridge, MA). The rs8192678 PGC-1α SNP was studied because of the association of this SNP with other diseases [22-30] and the A10398G mtDNA variant was chosen for study because of previous work that possibly links this SNP with decreased risk of PD [7-10]. The secondary PGC-1α SNPs were chosen based on previous work that studied the association of PGC-1α with Huntington's disease [31,32]. However, it was not possible to include one PGC-1α SNP from haplogroup 1 (rs17576121) in a single iPlex reaction with the other PGC-1α SNPs to be studied. Because the previous authors [31,32] did not show association between any of the haplogroup 1 SNPs and Huntington's disease we omitted this SNP from the study.

For purposes of quality control, a subset of samples were analysed for all polymorphisms using the polymerase chain reaction-restriction fragment length polymorphism (PCR-RFLP) method. Where possible, previously published primers sequences were used in this study [10,26,37]. Otherwise, primers were designed using the Primer3 program [38] and checked for sequence specificity against the NCBI database using primer-blast (http://www.ncbi.nlm.nih.gov/tools/primer-blast/). The sequences of these primers are listed in table S1 (see additional file 1.). PCR was performed using 1.25 U of AmpliTaq Gold (Applied Biosystems, Foster City, California, USA) with annealing at 54-59°C for 30s for detection of the *PGC-1α *SNPs and annealing at 50°C for 30s for the mtDNA SNP. The *PGC-1α *PCR products were digested using the restriction endonucleases listed in table S1 (see additional file 1 ) and the A10398G mtDNA polymorphism was analyzed by restriction digest as previously described [10]. All enzymes were obtained from New England Biolabs, Beverly, Massachusetts, USA).

### Statistical Methods

#### Association of *PGC-1α *and *ND3 *A10398G SNPs with risk of PD

To assess the association of the *PGC-1α *SNPs or the A10398G mtDNA variant with risk of PD, we fit conditional logistic regression models for case/control status, with adjustment for each of the 16 genotypes individually, and separately for early and late onset cases using the PD-ALL-EARLY and PD-ALL-LATE cohorts, respectively. The separate analysis of early (PD-ALL-EARLY) and late onset cases (PD-ALL-LATE) with their matched controls recognizes that there may be different genetic mechanisms that vary by age. However, we also jointly analyzed all matched cases and controls. We conditioned on age of PD onset for the PD cases and age of blood sampling for the controls in the models. Based on our sample sizes, we estimate (using the online calculator, http://prevention.cancer.gov/programs-resources/groups/b/software/power), that we have roughly 80% power to detect odds ratios for association between PD and genotype of 1.4-2.0 or larger (or 0.5-0.7 or smaller), based on two-sided 0.05 level tests. We also fit all 15 models with the ND3 A10398G SNP and each of the *PGC-1α *SNPs and their two-way interactions, again separately for the PD-ALL-EARLY and PD-ALL-LATE cohorts.

The validity of these models for assessment of association of SNP with risk of PD depends on the assumption that given age of PD onset, the genotype distribution is independent of the age of blood sampling for the PD cases. That is, if the PD cases available to be sampled have a different genotype distribution from those PD cases that are removed from the population by early death or other unavailability for blood sampling, then this analysis would not be valid. We partially tested this assumption by fitting logistic regression models for genotype for the PD cases only, with covariate adjustment for age of PD onset and age of blood sampling (both continuous and dichotomized at the sample median of five years post onset). We fit these models separately for the PD-ALL-EARLY and PD-ALL-LATE cohorts. Age of blood sampling, dichotomized at five years after diagnosis, was significantly associated with rs2970865 (p = 0.043) and with rs2970866 (p = 0.036) in the PD-ALL-LATE cohort. We used a 0.05 threshold, without any adjustment for multiple comparisons; this is conservative, as this will identify violations of the independence assumption more frequently than if had we used a lower significance level. Thus, we restricted the analysis of the association of these SNPs with risk of PD in this cohort to those PD subjects who provided samples within five years of diagnosis. This would diminish the bias due to sampling if these cases were more similar to the population of PD patients as a whole at the time of diagnosis.

#### Association of *PGC-1α *and *ND3 *A10398G SNPs with age-of-onset of PD

To assess the association of the SNPs with age-of-onset of PD, we used Cox regression models for age of onset of PD, with adjustment for the right truncation of age of PD onset by age at blood sampling. We fit this using the TPHREG procedure in SAS Version 9, after transforming this into a left-truncation setting via "time reversal." To adjust for the dependence between the age at blood sampling and the age of onset (over the observable region in which the first age necessarily follows the second), we included age at blood sampling in the model [39]. We did this in three ways: (1) including the continuous age as a covariate, (2) including tertiles of age at blood sampling as covariates, and (3) stratifying by tertiles of age at blood sampling. We applied this analysis to the PD-ALL-EARLY cohort, to the PD-ALL-LATE cohort, and to the PD-Cohort 1 cohort. We could not use the controls in this analysis (as censored for PD onset) because of their age-based selection. We estimate that we have 80% power to detect hazard ratios of 1.4-2.0 or larger (or 0.5-0.7 or smaller), using two-sided, 0.05 level tests; this is a rough calculation that does not take into account the adjustment for the truncation. We also used an alternative, cruder approach that obviated the need to treat the truncation by age at sampling. For this we used Fisher's exact test to assess the association between early (< 55) versus late (> 63) onset and genotype using the combined PD-ALL-EARLY and PD-ALL-LATE cohorts. Based on this analysis, we estimate that we will have roughly 80% power to detect differences in genotype prevalences among the early versus late groups of 10-20% or larger, using two-sided, 0.05 level tests.

#### Association of *PGC-1α *and *ND3 *A10398G SNPs with longevity

A genotype that appears to be associated with age of onset of PD might actually be associated with longevity, and not PD. We control for this in our analysis of risk of PD by matching on age. We do not control for this directly in our analysis of continuous age of onset of PD, in which we are not able to include the normal controls. To assess the association of the SNPs with longevity, we analyzed their associations with the age group of the controls, i.e., < 55 versus > 63. If any SNPs were associated with survival, we would expect to find a different distribution of genotypes among the younger controls versus the older controls. We tested this using two-sided Fisher's exact tests. We have approximately 80% power to detect differences in genotype prevalences among the two groups of 10-20% or larger, using two-sided, 0.05 level tests. We also assessed the association of SNPs with age using two-sided Wilcoxon or Kruskal-Wallis tests to compare the distributions of age between genotypes.

### Multiple comparisons and power

Because of our *a priori *interest in the rs8192678 SNP, we view the questions surrounding the rs8192678 SNP as being of primary interest. Because the remaining 15 SNPs did not have strong biological or clinical justification, we view their analysis, as well as the analysis of their interactions with rs8192678, as secondary and exploratory. Thus, we do not adjust any tests for multiple comparisons. We have provided approximations to the power that our study had for the analyses of interest. These are crude due to the complex nature of some of the analyses due to the adjustments for truncation. Nonetheless, they place the results in perspective and illustrate that in some cases, we could only have detected large associations.

## Results

### General Characteristics of participants

Two separate cohorts of PD DNA samples were obtained from the NINDS Human Genetics DNA and Cell Line Repository at the Coriell Institute for Medical Research (see table 1). The first, detection cohort of 179 PD samples (102 males and 77 females) were randomly selected from among the available Caucasian PD cases and initially screened for *PGC-1α *rs8192678 and *ND3 *A10398G polymorphisms. The mean age of PD onset for this selected cohort who provided blood samples at varying times after their onset was 58.8 in the male group and 57.2 in the female group, giving rise to an overall mean age of onset of 58 years in this group. An initial analysis indicated a non-significant trend towards an association of the rs8192678 *PGC-1α *polymorphism with age of onset of PD. Because of this, the second, or validation cohort of 197 Caucasian PD samples (also obtained from the NINDS Human Genetics DNA and Cell Repository at the Coriell Institute for Medical Research) was divided into an earlier age of onset group (35-55 years) and a later age of onset group (63-87 years). Overall, the mean age of onset for this second cohort, again who provided blood samples at varying times after PD onset, was 47.7 years for males and 45.7 years for females in the early-onset group and 74.7 for males and 76.1 for females in the late onset group. These age ranges were selected as they provided the maximum separation in ages between the earlier and later onset groups while still providing the desired numbers of samples from the available Caucasian PD samples at the NINDS Human Genetics DNA and Cell Line Repository at Coriell. None of the samples from the initial group were included in the second cohort. The control group was selected from the same repository to provide age-matched Caucasian controls for the samples in the early-onset and late-onset groups of the second cohort.

**Table 1:** Characteristics of the Sample Populations

	*Male*	*Female*	*Total Population*
Gender			
PD Cohort 1	102 (57.0%)	77 (43.0%)	179
PD Cohort 2	129 (64.8%)	70 (35.2%)	199
PD EARLY	63 (62.4%)	36 (35.6%)	99
PD LATE	66 (66.0%)	34 (34.0%)	100
PD-ALL	200 (60.8%)	129 (39.2%)	329
PD-EARLY-ALL	99 (57.9%)	72 (42.1%)	171
PD-LATE-ALL	101 (63.9%)	57 (36.1%)	158
Control	113 (65.3%)	60 (34.7%)	173
Control EARLY	49 (60.5%)	32 (39.5%)	81
Control LATE	64 (69.6%)	28 (30.4%)	92
			
Mean Age of Onset			
PD Cohort 1	58.8 (SD 10.6)	57.2 (SD 11.4)	58.0 (SD 11.0)
PD Cohort 2	61.5 (SD 14.7)	60.8 (SD 16.2)	61.2 (SD 15.2)
PD EARLY	47.7 (SD 5.4)	45.7 (SD 6.3)	47.0 (SD 5.8)
PD LATE	74.7 (SD 6.1)	76.1 (SD 5.4)	75.2 (SD 5.9)
PD-ALL	60.4 (SD 13.1)	58.6 (SD 15.0)	59.7 (SD 14.3)
PD-EARLY-ALL	47.6 (SD 5.3)	46.6 (SD 6.3)	47.1 (SD 5.8)
PD-LATE-ALL	72.9 (SD 6.2)	73.8 (SD 6.5)	73.4 (SD 6.4)
Control	NA	NA	NA
			
Mean Age at Sampling			
PD Cohort 1	63.8 (SD 9.8)	62.5 (SD 10.6)	63.2 (SD 10.2)
PD Cohort 2	66.2 (SD 14.6)	65.4 (SD 15.8)	65.8 (SD 15.0)
PD EARLY	52.1 (SD 6.1)	50.8 (SD 6.2)	51.7 (SD 6.1)
PD LATE	79.4 (SD 4.4)	80.5 (SD 4.3)	79.8 (SD 4.4)
PD-ALL	65.3 (SD 13.5)	63.6 (SD 14.3)	64.7 (SD 13.8)
PD-EARLY-ALL	52.9 (SD 6.2)	52.3 (SD 7.0)	52.6 (SD 6.6)
PD-LATE-ALL	77.4 (SD 5.4)	77.7 (SD 6.3)	77.5 (SD 5.9)
Control	61.1 (SD 5.9)	61.5 (SD 5.7)	61.3 (SD 5.8)
Control EARLY	47.9 (SD 5.7)	46.8 (SD 5.6)	47.3 (SD 5.7)
Control LATE	74.3 (SD 6.0)	76.3 (SD 5.8)	75.4 (SD 5.9)
			
Interval Between Onset and Sampling			
PD Cohort 1	5.4 (SD 4.6)	5.5 (SD 4.1)	5.4 (SD 4.8)
PD Cohort 2	4.7 (SD 2.5)	4.7 (SD 2.4)	4.7 (SD 2.5)
PD EARLY	4.6 (SD 2.4)	4.9 (SD 2.7)	4.7 (SD 2.5)
PD LATE	4.7 (SD 2.6)	4.5 (SD 2.2)	4.6 (SD 2.5)
PD-ALL	4.9 (SD 3.5)	5.2 (SD 3.7)	5.0 (SD 3.6)
PD-EARLY-ALL	5.3 (SD 3.9)	5.6 (SD 4.3)	5.5 (SD 4.1)
PD-LATE-ALL	4.5 (SD 3.0)	3.8 (SD 2.6)	4.2 (SD 2.8)
Control	NA	NA	NA

Data are presented for both cohorts and for each subgroup that was analyzed. The PD-ALL, PD- ALL-EARLY and PD-ALL-LATE groups represent subgroups drawn from both PD cohorts within restricted age parameters. Percentages are calculated from row data.

### Association of *PGC-1α *and *ND3 *A10398G genotypes with risk of Parkinson's disease

The frequencies of each SNP are shown in table 2. We did not detect a significant association between rs8192678 and risk among those with early onset PD (p = 0.37) or late onset PD (p = 0.84) or in the combined PD groups (p = 0.71). We also did not detect any significant interaction effects between any of the *PGC-1α *SNPs and the *ND3 *A10398G SNP for the risk of early or late onset PD.

**Table 2:** Frequency of each SNP within the Sample Populations

SNP	Alleles	PD-Cohort 1	PD-Cohort 2	PD-ALL	PD-EARLY-ALL	PD-LATE-ALL	All Controls	Control EARLY	Control LATE
rs12374310	GC	71 (41.5%)	78 (42.4%)	149 (42.0%)	72 (45.9%)	62 (41.3%)	81 (50.6%)	35 (49.3%)	46 (51.7%)
									
	CC	70 (40.9%)	77 (41.8%)	147 (41.4%)	55 (35.0%)	68 (45.4%)	49 (30.6%)	21 (29.6%)	28 (31.5%)
									
	GG	30 (17.6%)	29 (15.8%)	59 (16.6%)	30 (19.1%)	20 (13.3%)	30 (18.8%)	15 (21.1%)	15 (16.8%)

rs2946385	GT	79 (47.0%)	81 (45.5%)	160 (46.2%)	72 (47.4%)	65 (44.5%)	80 (50.3%)	38 (53.5%)	42 (47.7%)
									
	GG	60 (35.7%)	70 (39.3%)	130 (37.6%)	59 (38.8%)	54 (37.0%)	52 (32.7%)	21 (29.6%)	31 (35.2%)
									
	TT	29 (17.3%)	27 (15.2%)	56 (16.2%)	21 (13.8%)	27 (18.5%)	27 (17.0%)	12 (16.9%)	15 (17.1%)

rs2946386	GC	52 (30.8%)	77 (42.1%)	129 (36.5%)	55 (35.7%)	61 (40.7%)	49 (30.4%)	17 (23.6%)	32 (36.0%)
									
	CC	104 (61.5%)	93 (50.8%)	197 (55.9%)	88 (57.2%)	77 (51.3%)	98 (60.9%)	48 (66.7%)	50 (56.1%)
									
	GG	13 (7.7%)	13 (7.1%)	27 (7.6%)	11 (7.1%)	12 (8.0%)	14 (8.7%)	7 (9.7%)	7 (7.9%)

rs2970848	AG	76 (45.0%)	81 (44.3%)	157 (44.6%)	74 (47.7%)	60 (40.5%)	80 (50.0%)	32 (44.4%)	48 (54.5%)
									
	AA	80 (47.3%)	81 (44.3%)	161 (45.7%)	70 (45.2%)	69 (46.6%)	74 (46.3%)	37 (51.4%)	37 (42.0%)
									
	GG	13 (7.7%)	21 (11.4%)	34 (9.7%)	11 (7.1%)	19 (12.9%)	6 (3.7%)	3 (4.2%)	3 (3.5%)

rs2970855	AT	80 (47.0%)	93 (50.5%)	173 (48.9%)	78 (49.7%)	72 (48.3%)	78 (49.1%)	32 (45.1%)	46 (52.3%)
									
	TT	69 (40.6%)	63 (34.3%)	132 (37.3%)	60 (38.2%)	53 (35.6%)	59 (37.1%)	31 (43.7%)	28 (31.8%)
									
	AA	21 (12.4%)	28 (15.2%)	49 (13.8%)	19 (12.1%)	24 (16.1%)	22 (13.8%)	8 (11.3%)	14 (15.9%)

rs2970865	TC	56 (33.2%)	77 (41.6%)	133 (37.6%)	57 (36.5%)	62 (41.3%)	52 (32.3%)	18 (25.0%)	34 (38.2%)
									
	TT	105 (62.1%)	96 (51.9%)	201 (56.8%)	30 (57.7%)	79 (52.7%)	98 (60.9%)	48 (66.7%)	50 (56.2%)
									
	CC	8 (4.7%)	12 (6.5%)	20 (5.6%)	9 (5.8%)	9 (6.0%)	11 (6.8%)	6 (8.3%)	5 (5.6%)

rs2970866	AG	55 (32.2%)	75 (41.4%)	130 (37.0%)	54 (34.8%)	62 (41.9%)	50 (31.5%)	17 (23.6%)	33 (37.9%)
									
	AA	105 (61.4%)	94 (52.0%)	199 (56.5%)	90 (58.1%)	77 (52.0%)	97 (61.0%)	48 (66.7%)	49 (56.3%)
									
	GG	11 (6.4%)	12 (6.6%)	23 (6.5%)	11 (7.1%)	9 (6.1%)	12 (7.5%)	7 (9.7%)	5 (5.8%)

rs2970869	GA	55 (33.1%)	78 (42.2%)	133 (37.9%)	57 (37.0%)	62 (41.6%)	52 (32.1%)	18 (24.7%)	34 (38.2%)
									
	GG	104 (62.7%)	95 (51.3%)	199 (56.7%)	89 (57.8%)	78 (52.4%)	98 (60.5%)	48 (65.8%)	50 (56.2%)
									
	AA	7 (4.2%)	12 (6.5%)	19 (5.4%)	8 (5.2%)	9 (6.0%)	12 (7.4%)	7 (9.5%)	5 (5.6%)

rs2970870	TC	83 (49.4%)	87 (49.1%)	170 (49.3%)	77 (51.0%)	73 (50.0%)	72 (45.6%)	26 (36.6%)	46 (52.9%)
									
	TT	54 (32.1%)	58 (32.8%)	112 (32.4%)	47 (31.1%)	47 (31.1%)	57 (36.1%)	29 (40.9%)	28 (32.2%)
									
	CC	31 (18.5%)	32 (18.1%)	63 (18.3%)	27 (17.9%)	27 (17.9%)	29 (18.3%)	16 (22.5%)	13 (14.9%)

rs2970873	CT	40 (23.4%)	60 (33.0%)	100 (28.3%)	40 (25.3%)	48 (32.6%)	41 (25.8%)	15 (21.1%)	26 (29.6%)
									
	CC	126 (73.7%)	116 (63.7%)	242 (68.6%)	114 (72.2%)	93 (63.3%)	114 (71.7%)	53 (74.7%)	61 (69.3%)
									
	TT	5 (2.9%)	6 (3.3%)	11 (3.1%)	4 (2.5%)	6 (4.1%)	4 (2.5%)	3 (4.2%)	1 (1.1%)

rs4383605	TG	59 (35.1%)	73 (40.1%)	132 (37.7%)	55 (35.7%)	60 (40.5%)	59 (37.1%)	25 (35.2%)	34 (38.6%)
									
	TT	89 (53.0%)	97 (53.3%)	186 (53.1%)	84 (54.6%)	77 (52.0%)	83 (52.2%)	37 (52.1%)	46 (52.3%)
									
	GG	20 (11.9%)	12 (6.6%)	32 (9.2%)	15 (9.7%)	11 (7.4%)	17 (10.7%)	9 (12.7%)	8 (9.1%)

rs6821591	CT	92 (53.8%)	83 (46.3%)	175 (50.0%)	72 (46.8%)	74 (50.3%)	80 (50.3%)	40 (56.3%)	40 (45.4%)
									
	CC	39 (22.8%)	54 (30.2%)	93 (26.8%)	37 (24.0%)	47 (32.0%)	31 (19.5%)	7 (9.9%)	24 (27.3%)
									
	TT	39 (22.8%)	42 (23.5%)	81 (23.2%)	45 (29.2%)	26 (17.7%)	48 (30.2%)	24 (33.8%)	24 (27.3%)

rs7665116	CT	39 (22.8%)	43 (23.2%)	82 (23.0%)	41 (25.9%)	31 (20.7%)	56 (35.0%)	26 (36.1%)	30 (34.1%)
									
	TT	128 (74.9%)	140 (75.7%)	268 (75.3%)	114 (72.2%)	119 (79.3%)	102 (63.8%)	45 (62.5%)	57 (64.8%)
									
	CC	4 (2.3%)	2 (1.1%)	6 (1.7%)	3 (1.9%)	0 (0.0%)	2 (1.2%)	1 (1.4%)	1 (1.1%)

rs7695542	CT	17 (10.1%)	9 (4.9%)	26 (7.4%)	41 (26.0%)	31 (20.7%)	15 (9.3%)	8 (11.1%)	7 (7.9%)
									
	TT	151 (89.9%)	176 (95.1%)	327 (92.6%)	114 (72.1%)	119 (79.3%)	145 (90.1%)	64 (88.9%)	81 (91.0%)
									
	CC	0 (0.0%)	0 (0.0%)	0 (0.0%)	3 (1.9%)	0 (0.0%)	1 (0.6%)	0 (0.0%)	1 (1.1%)

rs8192678	GA	95 (53.0%)	88 (44.3%)	183 (48.4%)	89 (52.7%)	69 (43.7%)	83 (48.0%)	47 (58.0%)	36 (39.1%)
									
	GG	70 (39.2%)	93 (46.7%)	163 (43.1%)	66 (39.0%)	75 (47.5%)	73 (42.2%)	26 (32.1%)	47 (51.1%)
									
	AA	14 (7.8%)	18 (9.0%)	32 (8.5%)	14 (8.3%)	14 (8.9%)	17 (9.8%)	8 (9.9%)	9 (9.8%)

ND3 A10398G	A	143 (79.9%)	149 (75.6%)	292 (77.7%)	132 (79.0%)	119 (75.3%)	129 (74.6%)	60 (74.1%)	69 (75.0%)
									
	G	36 (20.1%)	48 (24.3%)	84 (22.3%)	35 (21.0%)	39 (24.7%)	44 (25.4%)	21 (25.9%)	23 (25.0%)

Data are presented for both cohorts and for each subgroup that was analyzed. The PD-ALL, PD- ALL-EARLY and PD-ALL-LATE groups represent sub-groups drawn from both PD cohorts within restricted age parameters. Percentages are calculated from column data.

However, our secondary analyses identified a significant association between rs2970848 and risk of late onset PD (p = 0.027), with high risk associated with the GG genotype (OR = 3.8 versus AA genotype and OR = 5.4 versus GA genotype). This SNP was not significantly associated with risk of early onset PD (p = 0.480), and this association did not quite reach statistical significance in the combined early and late groups (p = 0.063). We found a significant association between rs6821591 and risk of early onset PD (p = 0.029), with higher risk associated with the CC genotype (OR = 3.33 versus CT genotype and OR = 3.05 versus TT genotype). This SNP was not significantly associated with risk of late onset PD (p = 0.216) and in the combined early and late groups it was of borderline significance (p = 0.051).

### Association of *PGC-1α *and *ND3 *A10398G genotypes with age-of-onset of Parkinson's disease

Using the three types of Cox regression models for adjustment for dependent truncation due to the lag between blood sampling and PD onset, and using the various cohorts of PD cases described in the Methods section, we found no significant association between any *PGC-1α *SNP, or the *ND3 *A10398G SNP, with PD age-of-onset. Using the alternative, cruder, analysis of association between early versus late onset and genotype for the PD-EARLY-ALL and PD-LATE-ALL groups, we found a significant association between rs6821591and onset (p = 0.047), with 24% of early onset cases versus 32% of the late onset cases having the CC genotype, 46.8% of early onset cases versus 50.3% of late onset cases having the CT genotype, and 29.2% of early onset cases versus 17.7% of late onset cases having the TT genotype, as seen in table 2. This SNP was also found to be associated with longevity in controls (see below), and showed different genotype frequencies in the controls compared to the PD cases, as expected given the above finding of association of this SNP with risk of PD (which controls for age). In particular, among young controls, the frequency of the CC genotype was only 9.9%, in contrast to the 24% frequency among early onset PD cases. No association was seen between age of onset and the rs2970848 SNP. In addition, no significant interactions associated with age-of-onset of PD were observed between any of the other *PGC-1α *SNPs. A significant (p = 0.047) interaction between rs2970855 and the *ND3 *A10398G SNP was observed in the PD-Cohort 1 subset of samples, with the concurrence of the AT and A genotypes being associated with increased later onset. There was a trend toward this observation in the PD-ALL-EARLY subset (p = 0.076), but was not replicated across other PD sample groups. A significant (p = 0.044) interaction was also found between the *PGC-1α *rs7695542 and *ND3 *A10398G SNPs in the PD-ALL-LATE cohort, with the combined CT and A genotypes conferring earlier age of onset, but this was not replicated across other PD subgroups.

### Association of the *PGC-1α *rs8192678 and rs6821591 polymorphisms with longevity

A significant association between *PGC-1α *rs8192678 genotype and age group for control subjects was seen using a two-sided Fisher's exact test (p = 0.031). 58% of the 35-55 year old group had the AG genotype, versus 39.1% of the older controls; and 32.1% of the younger controls had had the GG genotype versus 51.1% of the older controls. The frequency of the AA genotype was comparable between the groups (9.8% and 9.9%). A significant association between *PGC-1α *rs6821591 genotype and age group for control subjects was also seen using a two-sided Fisher's exact test (p = 0.022). 9.9% of the younger controls had the CC genotype versus 27.3% of the older controls; 56.3% of the 35-55 year old group had the CT genotype, versus 45.4% of the older controls and 33.8% of the younger controls had the TT genotype versus 27.3% of the older controls. These distributions appear different from those in the PD cases, which is confirmed by the significance of the association of this SNP with PD risk reported above. No significant associations were detected between any SNP genotype and actual age of sampling for the control subjects within the younger cohort or within the older cohort.

## Discussion

Mitochondrial dysfunction and oxidative stress have been strongly linked to PD [12,13]. PGC-1α, which regulates mitochondrial biogenesis and expression of many antioxidant genes, plays an important role in protection against 1-methyl-4-phenyl-1,2,3,6-tetrahydropyridine (MPTP) toxicity [15], and expression of genes regulated by PGC-1α are reduced in substantia nigra neurons at early stages of PD [20]. These data led us to examine the association of common and potentially functionally relevant SNPs in the gene encoding PGC-1α with the risk of PD and age of onset of PD. A common mitochondrial complex I *ND3 *gene variant, 10398G, has been reported in some studies to be associated with decreased risk of PD, and thus we also examined the *PGC-1α *SNPs independently and in conjunction with the A10398G *ND3 *SNP for an association with risk of PD or age of onset of PD.

We did not find an association between the PGC-1α rs8192678 SNP and risk of PD or age-of-onset of PD. However, a significant association between the *PGC-1α *rs8192678 genotype and age group was found in our set of control subjects, with the GG genotype being significantly more common in older controls and the AG genotype more common in younger controls. This may suggest an association of the GG genotype with longevity. Potentially, this may be because the presence of the A allele has been associated with conditions detrimental to health such as diabetes, coronary artery disease, severe hypertension and alcohol consumption [40-46] whereas the GG genotype has been associated with lower fasting insulin levels [26]. Arguing against this explanation is our finding of a comparable frequency of the AA PGC-1a genotype the younger and older control groups. However, it is possible that the detrimental effect of the AA genotype may occur at an age even younger than that of our young control group (which was ages 35 - 55). An alternative interpretation of these data is that, rather than an effect on longevity, this *PGC-1α *variant could be associated with factors that influence the chances of volunteering to be a control subject at younger versus older ages.

There were no significant associations of the A10398G polymorphism with age group, either in the control population or the PD population. We also found no consistent association of the 10398G variant with risk of PD or age of onset of PD. Though this variant has been reported previously to be associated with a lower risk of PD, this finding has been variable across studies [7-11,47]. Differences in ethnicities may account for some of the apparent discrepancies, with a positive report in Caucasians [8] and in a Spanish population [48] and negative reports in Italian [9] and Greek populations [47]. However, other prior studies also had failed to detect a difference in analyses or sub-analyses of only Caucasian subjects [10,11,49].

Exploratory analyses identified that the rs2970848 GG variant may be associated with increased risk of late onset PD, but was not associated with the risk of early onset PD, or with the age of onset of PD when comparing frequencies in the later-onset and earlier-onset PD groups. The rs6821591 CC variant was associated with increased risk of earlier-onset PD (present at a higher frequency in earlier-onset PD patients compared to age-matched controls). Therefore, it may appear surprising that the CC variant was seen at a lower frequency in the earlier-onset PD patients (24%) compared to the frequency in the later-onset PD patients (34%). However, these observations are not contradictory. The finding of association with risk is within the younger groups and thus controls for age. That is, restricting to the younger groups, there is still a greater prevalence of the CC variant among PD patients compared to controls. Interestingly, one previous study [32] revealed an association of the rs6821591 SNP with delayed age of onset in Huntington's disease.

This is the first study to examine the association between *PGC-1α *single-nucleotide polymorphisms and PD. Variants of the rs8192678 *PGC-1α *polymorphism have previously been studied alongside polymorphisms in peroxisome proliferator-activated receptor delta (PPARD) and sirtuin 1 (SIRT1) in relation to Alzheimer's disease and were found not to be associated with AD in a Finnish case-control situation [50]. Two recent independent studies reported that *PGC-1α *haplotypes may be important modifiers for age of onset in Huntington Disease [31,32]. While the current results provide only limited evidence for a role for common PGC-1α variants in PD, a broader examination of *PGC-1α *SNPs is required before concluding whether or not other *PGC-1α *variants influence PD risk or age of onset.

## Conclusions

These data provide limited evidence of an association of certain PGC-1α SNPs with the risk or age of onset of PD, but the results are of borderline significance and as an exploratory study these findings require confirmation in separate cohorts. The association of the PGC-1α rs8192678 GG and rs6821591 CC variants with longevity deserves further study.

## Competing interests

The authors declare that they have no competing interests.

## Authors' contributions

JC participated in the design of the study, carried out molecular genetic studies, analyzed data and drafted the manuscript. SR carried out molecular genetic studies.

KZ carried out molecular genetic studies. RAB designed the statistical methods and performed statistical analyses. DKS designed the study, obtained samples and edited and approved the final manuscript. All authors read and approved the final manuscript.

## Pre-publication history

The pre-publication history for this paper can be accessed here:

http://www.biomedcentral.com/1471-2350/12/69/prepub

## Supplementary Material

Additional file 1 **List of primers and restriction enzymes used in this study**. Click here for file
